# Effects of Sedation with Medetomidine and Dexmedetomidine on Doppler Measurements of Ovarian Artery Blood Flow in Bitches

**DOI:** 10.3390/ani11020538

**Published:** 2021-02-19

**Authors:** Paloma Nicolás-Barceló, Martina Facchin, Fernando Martínez-Taboada, Rafael Barrera, José Ignacio Cristóbal, Mario Alberto González, Ángela Durán-Galea, Beatriz Macías-García, Francisco Javier Duque

**Affiliations:** 1 Veterinary Teaching Hospital, University of Extremadura, 10003 Cáceres, Spain; palomanicolas.1994@hotmail.com (P.N.-B.); martina.facchin@gmail.com (M.F.); rabacha@unex.es (R.B.); jignacristob@gmail.com (J.I.C.); marioalbgs@unex.es (M.A.G.); angeladg@hotmail.es (Á.D.-G.); javierduque@unex.es (F.J.D.); 2 Department of Veterinary Anaesthesia and Analgesia, School of Veterinary Science, Faculty of Science, The University of Sydney, Camperdown, NSW 3260, Australia; fer_m_taboada@hotmail.com

**Keywords:** medetomidine, dexmedetomidine, Doppler, ovarian artery, bitches

## Abstract

**Simple Summary:**

Alpha-2 agonists have widely been used in dogs as sedative and preanesthetic agents. These drugs have successfully been used in many different reproductive surgical procedures in dogs, including ovariectomy (OV) and ovariohysterectomy (OVH). The main problem associated with the alpha-2 agonists’ use is their dose-dependent cardiovascular effects and the changes induced on organ perfusion. Nowadays, the use of Doppler ultrasonography is the most commonly used method to assess physiological and pathological organ perfusion in veterinary medicine as it is a noninvasive technique. In bitches, Doppler ultrasonography has shown its usefulness to assess placental, ovarian and uterine blood flow during normal and abnormal pregnancy. We compared the ovarian artery flow velocity by duplex Doppler ultrasound before and after sedation with two commonly used alpha-2 agonists: medetomidine and dexmedetomidine. The results showed that the administration of medetomidine or dexmedetomidine induced an important decrease in blood flow velocities in the ovarian artery. Hence, their use could be indicated in surgeries to avoid excessive bleeding of the ovarian pedicle.

**Abstract:**

The aim was to evaluate if medetomidine and dexmedetomidine affected arterial ovarian blood flow in dogs. The dogs were randomly assigned to two different groups. In Group 1, medetomidine (10 µg/kg) was administered intramuscularly and, in Group 2, dexmedetomidine (5 µg/kg) was used. After a preliminary exam, arterial blood pressure (BP) was measured and a duplex Doppler ultrasonographic examination of both ovarian arteries was performed. Twenty minutes after the administration of medetomidine or dexmedetomidine, BP and ovarian Doppler ultrasonography were repeated. High quality tracings of ovarian artery flow velocity were obtained in all dogs and Doppler parameters: Peak Systolic Velocity (PSV), End Diastolic Velocity (EDV) and Resistive Index (RI) were measured before and after drug administration in the left (LO) and right (RO) ovaries. PSV and EDV values decreased significantly after drug administration (*p* < 0.05) compared to the non-sedated values, but no differences were found between the LO and RO (*p* > 0.05). The RI was not affected by drugs administration in neither of the groups studied (*p* > 0.05). In conclusion, the administration of medetomidine or dexmedetomidine causes a decrease in blood flow velocity in the ovarian artery and may be a good choice to avoid excessive bleeding prior surgeries in which ovariectomy.

## 1. Introduction

Alpha-2 agonists are widely used in dogs as a sedative [1] and preanesthetic agents [2]. Their analgesic properties also make them a simple and reliable drug as part of a balanced anaesthetic protocol to spare other anaesthetic agents in veterinary medicine [3,4,5]. Alpha-2 agonists have also the advantage of being easily antagonised by atipamezole in the case of a complication occurring [6].

Medetomidine is a racemic mixture of two optical enantiomers: dexmedetomidine and levomedetomidine. The dextro-rotary isomer (dexmedetomidine) is the active molecule and, when administered at half the dose of medetomidine, induces similar effects than those induced by it [7]. The levo-isomer (levomedetomidine) has classically been claimed to lack pharmacological activity, but it has been shown that the administration of high doses of levomedetomidine enhances bradycardia and reduces the sedative and analgesic effects associated with the administration of dexmedetomidine [8]. Due to this, the administration of dexmedetomidine alone may have some benefits over the administration of medetomidine. Both drugs have been successfully used in many different reproductive surgical procedures in dogs including ovariectomy (OV) and ovariohysterectomy (OVH) [9].

The main problem associated with the use of alpha-2 agonists is their dose-dependent cardiovascular effects [4,10,11]. Furthermore, changes in organ perfusion can take place, affecting differently specific organs. Medetomidine has been shown to render a lower perfusion index in the subcortical region of the brain, abdominal aorta, renal arteries, cranial mesenteric and celiac arteries in dogs [12]. On the other hand, dexmedetomidine has shown to decrease blood flow through arteriovenous anastomoses and decrease the perfusion of skin, kidney and brain [13].

Nowadays, Doppler ultrasonography is the most commonly used tool in veterinary medicine to assess organ perfusion and pathologies related to their blood supply, as it is a noninvasive technique. This technique is also widely used in cardio-circulatory diagnostic and reproductive medicine [14]. Doppler ultrasonography has been previously used to assess corpus luteum function, ovarian and uterine blood flow and to distinguish between luteal and follicular cysts in cattle [15,16]. In the bitch, Doppler ultrasonography has proven its usefulness in the assessment of placental, uterine and embryonic blood flow during normal and abnormal pregnancy [17,18] and also in the assessment of ovarian flow [19].

In recent years, there has been an increase in the surgical procedures presented to the veterinary reproductive clinic (OVH, OH and pyometra). All these procedures involve the removal of the ovaries and bleeding from the ovarian pedicle is the main complication associated to these surgeries, being intraoperatively haemorrhage linked to an increased mortality in this type of procedure [20].

## 2. Materials and Methods

For the present study, twenty healthy bitches of different breeds, weights and ages presented to the University of Extremadura Small Animal Hospital for elective OVH were used. The bitches were not recruited based on the stage of the oestrous cycle, but all dogs were sexually mature and had at least one heat before the procedure was scheduled. All animals used for the study were healthy, based on normal physical examination, haematology, basic biochemistry and a vaginal cytology sample using a vaginal swab; a vaginal smear for oestrus cycle assessment was performed and stained using Diff Quick^®^ (Micorptic S.L., Barcelona, Spain) stain.

Informed owner consent was obtained for all dogs and the study was approved by the Animal Ethics Committee of the University of Extremadura and it was performed in compliance with Spanish and European guidelines for research on animals (RD1201/2005 and ETS No. 170, respectively).

After the preliminary physical exam, all the dogs had the systolic arterial blood pressure (BP) measured (Ultrasound Blood Flow Detector MD4, Sonomed Ltd., Warsaw, Poland). Five consecutive BP values were obtained, the highest and lowest values being discarded, and the other three values were averaged and used for further statistical analysis.

A duplex Doppler ultrasonographic examination of the ovarian artery was performed before sedation to evaluate the basal blood flow velocity of each ovarian artery using a real-time scanner (Model HDI 5000, Philips Medical Systems, Bothell, WA, USA) equipped with a ATL 5-12 MHz linear transducer (ATL, Bothell, WA, USA). To optimize the performance and interpretation of Doppler images, some specific parameters for the spectral Doppler component (angle correction, spectral gain and gate size) and others specific to the colour Doppler component (colour gain and colour velocity scale) were adjusted by the operator in each dog. All ultrasound examinations were performed by the same skilled operator (F.J.D.) in a darkened room to avoid external stimuli. With the dogs in dorsal recumbency and without clipping the hair to minimise the stress, a generous amount of ultrasonic gel was applied before the abdominal scan. The left kidney was identified first, and the ovary was found caudally to the kidney. Once the ovary was identified, the colour-coded Doppler mode was used to locate the ovarian artery. In duplex Doppler mode, the optimal spectral graph of velocities during a cardiac cycle was obtained by adjusting the setting for detection of flow velocity for each examination. The width of the sample cursor was adjusted according to the diameter of the lumen of the scanned vessel. Then Peak Systolic Velocity (PSV), End Diastolic Velocity (EDV) and Resistive Index (RI) were calculated. RI was calculated according to the following formula:

RI = (PSV − EDV) / PSV(1)

Each value of the mentioned Doppler variables (PSV, EDV and RI) was measured three consecutive times from each vessel and then averaged; heart rate was also calculated (HR). The same measurements were repeated for the right ovary.

After the control ultrasound, the patients were randomly assigned to 2 different groups. In the first group (Group 1; *n* = 10) medetomidine (10 µg/kg) was administered and in the second group (Group 2) dexmedetomidine (5 µg/kg) was used. In both groups, the drugs were administered by deep intramuscular injection in the quadriceps muscle. After the administration of the sedative drugs, the dogs were placed in a darkened room for 20 min. After this time, the BP was measured again and the ovarian Doppler ultrasonic examination was repeated; this timing was chosen based on previous reports of our research group and other groups [21,22] as it is considered enough for both drugs to induce consistent sedation.

The data were first analysed for normality using the Kolmogorov–Smirnov’s test. In view of the Gaussian distribution of the data, the comparisons between the values before and after sedation and between the left (LO) and the tight (RO) ovaries were evaluated using a paired, one-tail *t*-test. Statistical significance was set at *p* < 0.05. Statistical analysis was performed using Minitab ver. 15.0 for Windows. Data are presented as mean ± standard error of the mean (SEM).

## 3. Results

No statistically significant differences were shown between groups in weight or age. In group 1 (*n* = 10) the dogs’ ages ranged between 12 and 60 months (32 ± 6 months) and their weights varied between 6.6 and 24.5 kg (15.8 ± 2.2 kg). In this group, based on the vaginal smears results, three bitches showed to be in proestrus, one in oestrus, four in diestrus, and two in anoestrus. In group 2 (*n* = 10) the ages varied between 24 and 60 months (35 ± 4 months) and the weight ranged between 7 and 37.2 kg (17 ± 3 kg). In group 2, two bitches were shown to be in proestrus, one in oestrus, four in diestrus and three in anoestrus.

In group 1, the HR before medetomidine administration was 104 ± 9 and decreased to 44 ± 8 after medetomidine administration (*p* < 0.01). Additionally, in Group 2, statistically significant differences in HR (beats per minute or bpm) were found before and after dexmedetomidine injection (99 ± 6 vs. 38 ± 5 respectively) (*p* < 0.001). BP in non-sedated patients in Group 1 was 145 ± 7 mmHg, dropping to 127 ± 5 mmHg after medetomidine administration (*p* < 0.05). In Group 2, blood pressure was 140 ± 3 mmHg prior to sedation, decreasing to 123 ± 6 mmHg after dexmedetomidine administration (*p* < 0.05). No statistically significant differences were found when medetomidine and dexmedetomidine treatments were compared for the HR and BP (*p* > 0.05).

**Figure 1:**
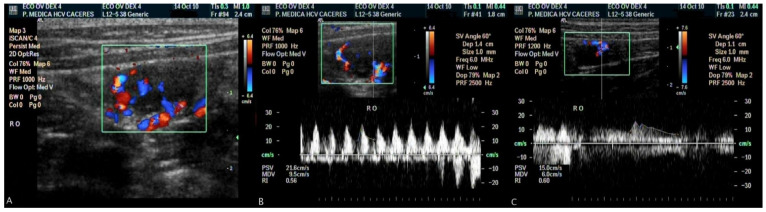
Colour flow Doppler image of intraovarian vascularization (**A**); spectral display of the Doppler signal from the right ovary before dexmedetomidine administration (**B**); spectral display of the same ovary after dexmedetomidine administration (**C**).

High quality tracings of ovarian artery flow velocity were obtained in all dogs and Doppler parameters (PSV, EDV and RI) were measured before and after medetomidine and dexmedetomidine administration in the LO and RO (Table 1 and Figure 1). PSV and EDV values decreased significantly after medetomidine and dexmedetomidine administration (*p* < 0.05) in the LO and RO. No statistically significant differences were found when LO and RO values were compared (*p* > 0.05). RI was not affected by drug administration in either of the groups studied (*p* > 0.05).

## 4. Discussion

The animals studied here were in different stages of the oestrous cycle and the two sedatives used influenced in a similar manner the monitored variables as previously described in the literature [23].

As previously reported, the administration of medetomidine or dexmedetomidine significantly decreased the HR [11,24,25]. The cardiovascular effects reported in the literature include not only bradycardia, but also arrhythmias, hypertension or hypotension, and reduced cardiac output [11]. In our study, bradycardia was observed in all animals, but no arrhythmias or hypotension were found. Unfortunately, cardiac output could not be measured. The two main causes of alpha-2-agonist-induced bradycardia are reduction in the sympathetic tone and increase in the systemic vascular resistance [11]; both causes can explain the results observed here. Hypotension can be explained by the stimulation of the central nervous system and the reduction in cardiac output as a consequence of the drop in HR and the increase in systemic vascular resistance and not by a direct depression of the myocardial contractility [26].

The BP was measured accurately following the guidelines reported in the literature [27]. The mean values measured were close to the parameters indicated by other authors [28]. According to Chalifoux, in dogs, the physiological blood pressure measured by Doppler is 145 ± 23 mmHg [28]. Any value higher or lower than this are considered hyper and hypotension, respectively. Hypertension may be due to stress or anxiety associated with the measurement itself [29,30,31]; or it may be secondary to medical conditions such as renal diseases, hyperadrenocorticism, hypothyroidism, diabetes mellitus, obesity, etc.; hypertension can also be idiopathic. As the physical examinations were normal in all bitches, the most plausible cause for the high BP in some non-sedated dogs included in our study might be stress. In cats, artefacts induced by anxiety (so-called “white coat effect”) can be reduced up to 20 mmHg by acclimatization and it is likely that similar results may be expected in dogs [31]. For that reason, and trying to avoid this stress effect, the measurements took place in a quiet and darkened room after a 5–10-min period of acclimatization.

In our study, a significant decrease in BP was observed only after dexmedetomidine use. Medetomidine did not significantly influence blood pressure. This result agrees with various studies published on the use of medetomidine in dogs reporting that significant hypotension during the study was not observed, but a general trend of decreasing blood pressure towards baseline or normal levels has been observed [25,32,33,34,35,36,37]. The hypotension caused by dexmedetomidine administration could be explained because of the higher affinity of dexmedetomidine to alpha-2-adrenoreceptor when compared to medetomidine [38]. However, dexmedetomidine is not a pure alpha-2-agonist as it is also able to trigger the activation of noradrenergic imidazoline receptors [39]. The stimulation of these receptors mediates a central hypotensive and anti-arrhythmogenic action, thus explaining the observed effect of dexmedetomidine on blood pressure.

Regarding ovarian artery blood flow velocity measurements, in 1989, Jedruch and others [40] reported the increase in uterine contractility in bitches sedated with medetomidine, also suggesting an effect of this drug in the reproductive tract [40]. Aside from this report, there is not further information about the effect these sedatives have on ovarian blood flow velocity and resistive index in dogs.

The bitches used in this study were in different stages of the oestrous cycle which were verified with a vaginal cytology and compared with the published bibliography [19]. Köster and others compared the vaginal cytology and progesterone quantity with the ovarian artery blood flow velocity measured with pulsed Doppler ultrasonography. We analysed the blood flow velocity in the different phases of oestrous cycle: during the oestrous phase, the blood flow was faster than during anoestrous (Table S1) and these results were similar to Köster’s study [19]. In mammals, during the oestrous phase, there are hemodynamic changes that are essential for follicular modelling and for the function of reproductive tissues [41,42]. A moderate increase in ovarian vascularization during the follicular phase, which is attributed to the influence of various angiogenetic growth factors stimulated by gonadotropins and produced by the proliferating cells of the follicular wall [41,43]. In some of our dogs, PSV and EDV were higher than the values reported by Köster [19]. The main difference between our study and previous studies is the use of a more modern ultrasound machine. The angle between the Doppler stream and the vascular segment used in this study was electronically kept lower than 60° and this gives a more accurate measurement since higher angles may give false lower reading. Additionally, Köster used laboratory Beagle dogs for his study, while we used clinical dogs (so stress might be responsible for the high BP and blood flow velocities).

The blood flow was similar in the right and left ovaries during the various oestrous phases, as previously reported by Köster et al. [19]. In the bitches studied here, blood flow was detected even during anoestrous, while Köster et al. were not able to detect the blood flow in 14 of 22 dogs at 70 days after ovulation [19]. This might be because the dogs were in a premature anoestrous or because the ultrasound scanner used was more modern and potent. Some individual variations may also be attributed to an inaccurate definition of the insonation angle between Doppler stream and alignment of the arterial segment.

RI is fundamentally related to the systemic vascular resistance. In this study, no significant changes in RI were shown; this could be attributed to the fact that medetomidine and dexmedetomidine do not exert any effect on RI in the ovary artery in bitches, or that the ultrasound examination was performed in the second phase of action of medetomidine when the effect on the central nervous system causes hypotension and decreases the cardiac output [11]. Cardiovascular effects of medetomidine and dexmedetomidine are described in two phases: the initial peripheral phase is characterized by vasoconstriction, increased blood pressure, and reflex bradycardia, while the subsequent central phase is characterized by decreased sympathetic tone, heart rate and blood pressure. As reported in the literature, alpha-2-agonists administration decreases cardiac output but blood flow to the heart, brain, and kidneys is maintained by the redistribution of flow from less vital organs and tissues [13]. Alpha-2-agonist effect on the ovarian artery in dogs has not been studied previously. The decrease in PSV and EDV in the ovarian artery observed in this study after medetomidine and dexmedetomidine administration is probably due to a redistribution of blood to vital organs at the expense of less vital organs, such as those of reproductive tract. The bitches examined were in different stages of the oestrous cycle, but this did not significantly affect the results: in each bitch there was a significant decrease of PSV and EDV despite the phase of the oestrus cycle.

Miño et al. studied in 2008 the effect of medetomidine (30 µg/kg) on the Doppler variables of the major abdominal arteries in normal dogs [12]. After the administration of the drug, a decrease in PSV and EDV was observed, but RI also remained unchanged. These results are similar to those obtained in our study, and they are probably due to a decreased cardiac output. Our results and those reported by Miño et al. [12] may correlate with the dose used. According to Pypensop and Verstegen [37], medetomidine doses under 2 µg/kg exert minimal cardiovascular effects. However, maximal cardiovascular effect was obtained at 5 µg/kg and further dose increases only lengthened the duration of the cardiovascular effects [32]. In 2009, Araujo et al. examined the effects of xylazine and detomidine on iliac artery perfusion and the reproductive tract of pony mares and heifers with duplex Doppler ultrasonography, and they observed a decrease in blood flow velocity in the internal iliac arteries, but no effect on the ovarian or endometrial perfusion was detected (probably due to protective mechanism of blood flow regulation) [44].

## 5. Conclusions

In conclusion, the administration of medetomidine or dexmedetomidine at equipotent doses causes a decrease in blood flow velocity in the ovarian artery. A reduced perfusion could be interesting when removing the ovaries to reduce the risk of haemorrhage. It is unknown if this decrease in ovarian perfusion may be detrimental when these drugs are used in the sedation of breeding or pregnant animals. For this reason, further studies investigating these effects are needed. Finally, sedation with medetomidine or dexmedetomidine affects the ovarian blood flow and does not allow for an accurate measurement of the ovarian artery blood flow.

## Figures and Tables

**Table 1:** Group 1 and 2 before and after sedation (*n* = 10 for each group).

Doppler Parameters		Before Administration				After Administration			
		Group 1		Group 2		Group 1		Group 2	
		Mean	SEM	Mean	SEM	Mean	SEM	Mean	SEM
**RO**	**PSV**	20.79	1.44	19.50	1.54	11.97	1.6	12.50	1.53
	**EDV**	8.09	0.78	8.20	1.05	5.15	0.88	5.65	0.76
	**RI**	0.61	0.03	0.59	0.03	0.58	0.02	0.55	0.02
**LO**	**PSV**	20.30	3.14	19.92	1.99	10.94	1.26	13.03	1.41
	**EDV**	6.72	0.8	7.40	0.08	4.20	0.52	5.82	0.98
	**RI**	0.66	0.02	0.63	0.02	0.62	0.01	0.58	0.03

PSV: Peak systolic velocity; EDV: End diastolic velocity; RI: resistive index; RO: right ovary; LO: left ovary.

## Data Availability

Data are available from the corresponding author upon reasonable request.

## Supplementary Materials

The following are available online at https://www.mdpi.com/2076-2615/11/2/538/s1, Table S1: Ovarian flow velocities divided by the oestrus cycle stage prior sedation.
