# Supplementary material for: Effects of Sedation with Medetomidine and Dexmedetomidine on Doppler Measurements of Ovarian Artery Blood Flow in Bitches
Source: Animals (Basel). 2021 Feb 19;11(2):538. doi: 10.3390/ani11020538 (PMC7922352; doi:10.3390/ani11020538)
Supplement: Supplementary file 1 [file animals-11-00538-s001.pdf]

animals

MDPI

Article

Supplementary File: Effects of Sedation with Medetomidine and Dexmedetomidine on Doppler Measurements of Ovarian Artery Blood Flow in Bitches

Paloma Nicolás-Barceló [1,†], Martina Facchin [1,†], Fernando Martínez-Taboada [2], Rafael Barrera [1], José Ignacio Cristóbal [1], Mario Alberto González [1], Ángela Durán-Galea [1], Beatriz Macías-García [1,*] and Francisco Javier Duque [1]

Table S1. Ovarian flow velocities divided by the oestrus cycle stage prior sedation.

	Proestrous *n* = 5	Oestrus *n* = 2	Diestrus *n* = 8	Anoestrus *n* = 5
**PSV (RO)**	20.8 ± 0.7	31.3 ± 0.4	19.9 ± 0.6	15.3 ± 0.6
**PSV (LO)**	19.05 ± 2.2	41.2 ± 5.5	18.8 ± 0.7	14.8 ± 0.3
**EDV (RO)**	8.9 ± 0.4	13.4 ± 1.7	8.07 ± 0.7	5.3 ± 0.9
**EDV (LO)**	8.06 ± 1.3	10.8 ± 0.8	6.8 ± 0.6	4.9 ± 0.4
**RI (RO)**	0.56 ± 0.01	0.57 ± 0.06	0.6 ± 0.02	0.6 ± 0.06
**RI (LO)**	0.58 ± 0.03	0.74 ± 0.01	0.6 ± 0.02	0.7 ± 0.02

All the dogs included in the present study (*n* = 20) were grouped by their oesrus cycle stage, and the descrisptive statistics Mean ± SEM. Are presented. PSV: Peak systolic velocity; EDV: End diastolic velocity; RI: resistive index.
